# The Effect of Consumer Involvement in Light Lamb Meat on Behavior, Sensory Perception, and Health-Related Concerns

**DOI:** 10.3390/nu11061200

**Published:** 2019-05-28

**Authors:** Guillermo Ripoll, Begoña Panea

**Affiliations:** 1 Centro de Investigación y Tecnología Agroalimentaria de Aragón (CITA), Avda. Montañana, 930, 50059 Zaragoza, Spain; bpanea@aragon.es; 2 Instituto Agroalimentario de Aragón–IA2 (CITA-Universidad de Zaragoza), C/Miguel Servet, 177, 50013 Zaragoza, Spain

**Keywords:** beliefs, concerns, attitudes, quality cues, choice, preferences

## Abstract

Involvement can explain behavioral consequences, such as consumer decision-making and consumption. The first aim of this study is to identify the profiles of consumers based on their involvement in light lamb meat. The second aim is to study the influence of involvement on consumers’ attitudes, behaviors, beliefs, preferences, quality cues, and sensory perception regarding light lamb meat. Two consumer profiles are identified. The first cluster includes consumers who enjoyed eating light lamb meat, were conscious of their self-image, and perceived the consequences of poor choices; these consumers also perceived the probability of making an incorrect choice as high. The second cluster includes consumers who truly loved eating lamb meat, were also conscious of their self-image, and perceived the consequences of poor choices; however, these consumers were confident in not making incorrect choices. Although both involvement-based profiles showed high involvement in light lamb meat, it can be concluded that the second cluster had a higher involvement. In general, the involvement-based profiles did not influence health-related attitudes, preferences, or sensory perceptions of light lamb meat, while beliefs, behavior and quality cues were influenced by involvement.

## 1. Introduction

Sheep farming systems use marginal areas of Mediterranean Europe and are mostly considered to be High Nature Value farmland [1]. In addition, these farming systems are multifunctional [2] and provide meat products with extrinsic qualities, such as local origin or production by environmentally friendly methods [3]. However, the number of farms in most European Mediterranean regions has suffered a sharp decline in the last few decades [4]. This decline is a direct consequence of a steady diminution of lamb meat consumption over the years; the consumption in Spain was 2.8 kg/head in 2004, 2.4 kg/head in 2009, and decreased dramatically to 1.3 kg/head in 2017. The consumption of light lamb in Aragón, the Spanish region where this study was carried out, was 7.5, 6.3, and 2.8 kg/head in 2004, 2009, and 2017, respectively [5]. Although the light lamb consumption in Aragón has also been reduced in the last few years, the consumption in 2017 was more than twice of the consumption of Spain. Aragón is known for the production of the ‘Ternasco de Aragón’ Protected Geographical Indication (PGI) lamb [6,7]. This kind of light lamb is suckled for at least 50 days, and then it is fed concentrate and straw to an age of between 70 and 90 days. Consequently, the chilled carcass weight ranges from 8 kg to 12.5 kg. Lamb meat from this production system is characterized by the pale pink color of the muscle and by white subcutaneous and perirenal fat. Lamb meat is increasingly perceived as a traditional product, mainly consumed in celebrations and social events. However, this type of consumption is particularly uncommon in young people, who normally do not have cooking skills and are not willing to pay high prices [7]. In 2017, the price of light lamb in Spain was 10.9 €/kg, while the prices of chicken and pork were 4.1 and 5.9 €/kg, respectively [8]. However, Argemí-Armengol et al. (2019) reported that credence cues claiming health issues influenced their willingness to pay for pork rather than consumers’ culinary skills [9]. The differentiation of lamb meat through consumer-led development and the further segmentation of markets is often pointed out as a relevant strategy to increase the consumption of light lamb meat [10]. Thus, involvement is a key factor in designing marketing strategies to increase the consumption of light lamb meat. It can be deduced that the future consumption of light lamb meat will depend greatly on the diversification and advertising of lamb meat products to reach different consumers.

This study is focused on consumer involvement in light lamb meat to characterize consumer profiles and analyze the relevant differences between them. This approach fits the model established by Laurent and Kapferer [11] and oriented by Verbeke and Vackier [12] to the segmentation of markets for specific (consumer-led) meat products [11,12,13,14]. To the best of our knowledge, few researchers have studied consumer involvement in meat, and none have focused their attention on light lamb meat and its effects. The results of this study will help the industry better profile consumers within commercial strategies by using relevant intrinsic and extrinsic quality cues, health claims, beliefs, and preferences. This information fills the gap between consumer research and the light lamb meat market because involvement is a psychological concept with a straightforward marketing application [15,16,17] explaining consumers’ purchasing decisions [12,18,19]. Therefore, the general objective of the research is to provide information to the meat market to find consumer-oriented strategies to enhance the consumption of light lamb meat. In addition to this, involvement is related to high consumption of healthy foods, such as vegetables and fruit [20]. Moreover, involvement can contribute to the adoption of healthy eating habits, because involvement is an important determinant for making healthy food choices [21]. As suggested by Van Loo et al. [22], for informational food policy actions relating to health, researchers need to address and trigger issues that are relevant to the target consumers. Therefore, involvement appears to be a key trigger to increase healthy eating.

## 2. Theoretical Background

Product involvement is defined as the degree to which a product is centrally related to a consumer’s value system and refers to feelings of interest, concern, and enthusiasm towards products [18]. Highly involved consumers enjoy searching for and processing information to modify their choice behavior [22]. The concept of involvement in relation to products comes from psychology, but much of the interest in this concept has been generated by marketing [23]. There is interest in involvement because it can explain behavioral consequences, such as consumer decision-making and consumption decisions, and it determines the importance of food purchasing decisions [12,18,24]. There are two forms of involvement according to Richins and Bloch [25]. Our study is focused on “enduring involvement,” which is the level of interest in a product that does not change greatly over time. “Situational involvement,” on the other hand, represents a temporary increase in interest due to short-term changes in circumstances and is not studied here. Involvement includes the feelings of consumers in relation to a product, such as attention, curiosity and enthusiasm [26]. Consequently, involvement connects the product and the consumer, so it depends on the characteristics of both the product and the consumer [27]. According to Espejel et al. [28], consumer involvement with a product is defined as the degree of importance that the consumer attaches to the product, given the consumer’s inherent needs, values, and interests, and is evoked by consumer-related stimuli that help the consumer overcome specific situations or reach certain aims. Therefore, involvement in a food product is determined by the level of perceived personal importance evoked by this food linked to a situation-specific goal. In the literature, consumers were frequently classified as highly involved or less involved consumers in relation to a certain product [28]. Highly involved consumers are more motivated to invest cognitive effort into the decision-making process, thus shaping the importance of their food purchase decisions [29]. Laurent and Kapferer [11] posit the following characteristics of involvement: the perceived importance of the product, the hedonic value of the product or the ability to provide pleasure, the symbolic value attributed to the product by the consumer, the importance of risk and negative consequences in the case of a poor choice, and the risk probability of making a mistake. Since consumer involvement is a multidimensional construct [11,12,14], all aspects of involvement must be simultaneously taken into account to identify a full consumer profile. Segmenting consumers according to their involvement is interesting because highly involved consumers are more receptive to advertising messages than less involved consumers.

Food products are usually considered low-involvement products, because they are purchased frequently and often at low prices [18,27]. Consequently, examples of low-involvement food products are coffee, bread [30], and fruit juice [31]. These food products have low potential to reflect their own image of the consumer, low prices, and low social pressure to make consumers be content with the choice of a product [12]. However, this interpretation does not take into account all aspects of involvement, such as the symbolic value due to a negative image, or even the importance of risk and risk probability. These features of involvement are important in relation to food products traditionally considered to be high-involvement food products, such as wine [32], olive oil [31], and fresh mussels [33]. These products have high prices and are complex, so the risk to fail when purchasing is also high [34]. Protected Designation of Origin (PDO) food products are also high-involvement products because both the brand and symbolic side of consumption play a relevant role [28]. According to Verbeke and Vackier [12], the symbolic value of red meat is low because it is unable to spread the image of consumer and manage impressions. On the other hand, Espejel, Fandos, and Flavián [28] argue that a consumer may be greatly involved in Protected Designation of Origin (PDO) foods because the symbolism plays a relevant role in their consumption. Another factor that can increase the involvement in meat, together with PDO, is familiarity. Borgogno, Favotto, Corazzin, Cardello, and Piasentier [14] reported that consumers who were highly familiar with and knowledgeable about sheep meat were more involved than less-familiar consumers. Highly familiar consumers give more hedonic and symbolic value scores together with product importance than other consumers. Aragón is notable for its high consumption of light lambs. This meat is more expensive than pork and chicken, so light lamb is a valuable product for this region [10,35]. Price is an important indicator of involvement because when the risk of making a mistake at the time of purchase is high, involvement is also high. In addition, consumers show a higher level of involvement with the Protected Designation of Origin food products [28].

As shown previously, involvement can explain the decision-making process and its impact on meat consumption decisions [12]. Using this framework model, the industry can translate knowledge about different involvement profiles into their commercial strategies by using relevant intrinsic and extrinsic quality cues and distribution channels. Verbeke and Vackier [12] studied how consumers, differing in their involvement in fresh meat, had different attitudes, concerns, and behaviors toward meat.

According to the literature, a high consumption of red meat is related to dietary colorectal cancer, although the weak association and confounding effects of other dietary and lifestyle factors make the dose-response effect unclear [36]. However, the International Agency for Research on Cancer (IARC) published a report [37] that classified red meat as probably carcinogenic to humans. This report and the following monograph [38], in addition to some other meat safety crises in Europe, have had a negative effect on the perception of meat. The reaction of consumers toward this negative reporting has involved changes in their attitudes, concerns, beliefs, and behaviors toward meat products [28]. In fact, highly involved consumers of beef were the same consumers who decreased their consumption due to the bovine spongiform encephalopathy (BSE) crisis [13]. As a result of these attitudes, this study is focused on consumer concerns regarding health topics related to light lamb meat, such as concerns with cholesterol and colorectal cancer. Other potential effects of involvement include the frequency of consumption and behavioral intentions. These two aspects of behavior concur with those studied by Verbeke and Vackier [12]. According to the study by Schulz and Hamm [13], consumers highly involved in beef were willing to pay more for beef consumption-related services, more often patronizing the niche market and disapproving of beef bargains and discounts. The acceptance of food products is mediated by expectations and previous experiences, among many other factors [39]. In addition, there is also the factor of belief, or credence quality, for which information is not available during purchasing and even consumption [40]. Consumers, therefore, require knowledge and practical experience to evaluate the quality of a product. This concept of belief, or credence quality, is similar to the concept of the quality attribute defined by Steenkamp [41]. In many cases, the consumer trusts information delivered by mass media, advertisements, word of mouth, third-party judgements, the seller’s credentials, etc. [42,43]. Highly involved consumers make an effort to gain knowledge about a product [32] and are, therefore, less influenced by common beliefs about light lamb meat at the time of purchase than related intrinsic quality cues (e.g., the color of meat and fat) including desirable or undesirable characteristics, such as lamb age or unpleasant flavor. However, involvement also implies stronger beliefs about product attributes [44]. Common credence attributes of meat studied in the literature include origin, animal welfare, production system/feeding, health/nutrition, and environmental issues [45]. Most of the credence attributes or beliefs about light lamb try to relate production system characteristics with meat quality [46]. Consequently, this study aimed to investigate consumer agreement with several suggested beliefs regarding light lamb meat, and the influence of their level of involvement on these beliefs.

Product quality cues are often categorized as either intrinsic or extrinsic. Intrinsic cues are the physical characteristics of a product, whereas extrinsic cues are product-related, but not a part of the physical product itself [41,47]. Both perceived intrinsic and extrinsic quality cues build product quality as a multidimensional construct [48] that serves as a surrogate indicator of quality to consumers/shoppers [49]. These quality cues, therefore, elicit consumer interest in the product [50] and are important because they reinforce the feelings of pleasure gained by highly involved consumers by means of purchase and consumption [28]. Some authors reported that consumers with high involvement respond to intrinsic, rather than extrinsic, cues [32], whereas other authors reported the contrary, i.e., that consumers with more product knowledge and involvement rely more on extrinsic cues [51,52].

Extrinsic cues have been shown in the literature to affect overall product acceptability and to alter the sensory experience of taste [53]. On the other hand, less involved consumers do not alter their sensory acceptance when the quality characteristics of the food product change [54]. In addition, less involved consumers are more dependent on the environment in their evaluation of sensory characteristics. In contrast, highly involved consumers can perceive differences in the sensory properties of products [19,50]. Furthermore, a consumer’s background influences his or her sensory perception of food [55]. Lahne et al. [56], also demonstrated that consumer preference was significantly affected by involvement when tasting cheese. Consumer involvement may, therefore, modify the perception of the sensory characteristics of food products [19]. Verbeke and Vackier [12] studied how consumer profiles differing in their involvement in fresh meat was reflected in their attitudes, concerns, and behaviors; these authors concluded that red meat has a low symbolic value because it is unable to develop its consumer image and manage impressions.

**Figure 1:**
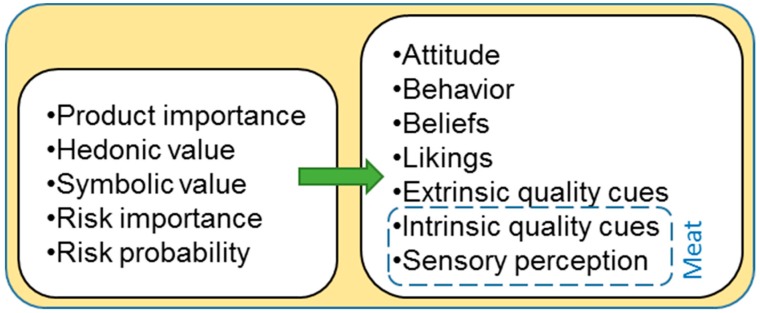
Framework for the study of involvement in light lamb meat based on the theoretical model proposed by Verbecke and Vackier (2004).

This research is based on previous work, which shows that involvement leads to stronger beliefs about product attributes [44], since highly involved consumers make more effort to gain knowledge about a product [32]. Therefore, it is likely that highly informed consumers would be less influenced by common beliefs about light lamb meat than by related intrinsic quality cues at the time of purchase. Given this conceptual framework (Figure 1), this research is based on seven hypotheses:

**H1.**  Consumers in Aragon have a high involvement in light lamb meat, following the theoretical background in Laurent and Kapferer [11].

**H2.**  Consumers can be categorized according to their level of involvement in light lamb meat.

**H3.**  Highly involved consumers have fewer concerns related to health than lowly involved consumers.

**H4.**  (I) Highly involved consumers do not modify their past or future frequency of consumption, while less involved consumers do, and (II) less involved consumers prefer the convenience format of light lamb meat.

**H5.**  (I) Consumers agree with the proposed beliefs, and (II) highly involved consumers show a higher degree of agreement with the proposed beliefs than less involved consumers.

**H6.**  (I) Highly involved consumers trust extrinsic cues more than intrinsic cues, and (II) highly involved consumers give more importance to quality cues than less involved consumers.

**H7.**  The sensory perception of light lamb meat is modified by the level of involvement.

## 3. Materials and Methods

### 3.1. Recruitment

A total of 100 participants were recruited by advertisements in social media and email, at associations, and at public events. People were eligible to participate unless they had cultural, religious or ethical issues with lamb consumption. In addition, it was mandatory that participants were light lamb meat consumers responsible for food purchasing in their households. This study was conducted according to the Declaration of Helsinki for studies on human subjects, and the protocol was approved by the Research Center Review Board. Prior to participation, all participants were explained the experimental procedure and gave their written informed consent. Participants attended a single session for approximately 40 min, and they were rewarded with a bottle of olive oil (total value: 2.31 €) as compensation.

### 3.2. Meat Samples and Preparation

Ten light lambs from the “Ternasco de Aragón” Protected Geographical Indication were selected from a homogeneous production batch of the Ojinegra de Teruel breed and fed concentrates and barley straw. Lambs were slaughtered following standard commercial procedures according to European norms for the protection of animals at the time of killing [57]. The cold carcasses of the light lambs were weighted 11.1 kg ± 0.31 kg (mean ± s.e.). Both the *longissimus thoracis* and *lumborum* muscles (LTL) of each carcass were extracted and vacuum-packaged together. The plastic bags (Marmelot Bvba, Retie, Belgium) were made of a 90 microns thick foil formed by layers of polyamide (20 microns) and polyethylene (70 microns). Samples were cooked in a Selecta Precisdig thermostatic water bath (J. P. Selecta, Barcelona, Spain) at 75 °C for 15 h. Once the cooking process was completed, the pouches were removed from the water bath and kept at room temperature for 2 h until the samples were prepared. Next, any external connective tissue was trimmed off each loin, which was then cut into 10 cubes of approximately 2 × 2 × 1 cm. The cubes were wrapped in codified aluminum paper and stored at 60 °C until the tasting. Two cubes from the same lamb corresponding to each of the two LTL were paired.

### 3.3. Consumer Tests

Ten sessions each involving 10 different consumers were developed, so 100 consumers aged 21 to 82 years (mean = 48 years ± 12.9) participated. A total of 48% of consumers were women, 66% had children, and 17% studied in primary school, 40% studied in secondary school, and 43% completed higher studies. Consumers were instructed to avoid eating, drinking anything other than water, chewing gum, and smoking 1 h prior to the test. While the subjects were not trained, they received both verbal and written instructions before every session. Consumers were first required to attend a session where two blind samples of meat were simultaneously presented. Because intramuscular fat percentage, drip loss and tenderness of LTL may vary from the caudal to cranial sides [58,59,60], thereby affecting sensory scores, two samples were presented to achieve robust scores while avoiding effects due to intramuscle location. In addition, both samples were from the same lamb to avoid an animal effect, and the order of the presentation of the samples was controlled. After tasting each sample, participants were asked to rate the acceptability of juiciness, tenderness, and flavor on a 9-point hedonic scale ranging from 1 (dislike extremely) to 9 (like extremely). The scores of both meat samples were averaged. Spring water (Veri, Aguas de San Martín de Veri, S.A, Bisaurri, Spain) and unsalted crackers (Hacendado, Puebla de Farnals, Spain) were provided for participant palate cleansing. Next, the participants were requested to fill out a questionnaire.

### 3.4. Questionnaire

Consumer involvement was measured using a 15-item scale comprised of 5 subdimensions developed by Laurent and Kapferer [11]. These items are shown in Table 1. Each item was scored on a seven-point Likert (interval) scale, ranging from 1 = “totally disagree” to 7 = “fully agree”.

Consumers were also asked about their attitudes and concerns on topics related to health and meat consumption (the use of antibiotics/hormones to raise lambs, the presence of fat and cholesterol in light lamb meat, and the relation between red meat consumption and colorectal cancer), and their purchasing and consumption behavior (frequency of consumption and behavioral intention). These questions are the same as those studied by Verbeke and Vackier [12], but ‘fresh meat’ was replaced with ‘light lamb meat’. Other questions about light lamb meat-related beliefs and light lamb meat preferences (for the color of meat and fat) were also proposed [10]. All these questions were scored on a seven-point Likert (interval) scale, ranging from 1 = “totally disagree” to 7 = “fully agree”, as shown in Table 2.

The level of importance that consumers ascribe to different quality product cues was assessed using a 5-point scale, in which 1 = “none or very little importance”, 2 = “little importance”, 3 = “average importance”, 4 = “quite a lot of importance”, and 5 = “great importance” [61]. The specific cues were selected from the literature and included both intrinsic and extrinsic cues [61,62,63,64]. Additional questions about purchasing behavior and questions concerning sociodemographic data (age, gender, presence of children at home, educational level, living environment, and frequency of light lamb meat consumption) were also included.

The answers in each section of the questionnaire used different scales according to the proposed methodologies [11,12], in order to easily compare results with past studies’ results.

### 3.5. Statistical Analysis

Data analysis was performed using XLStat v 2014.3.05 (Addinsoft, Barcelona, Spain), and data analysis procedures were reported by Verbeke and Vackier [12]. A factor analysis using principal components with Pearson correlation as the extraction method was performed to assess the involvement profiles. No rotation was used. Cronbach’s alpha was used to test the reliability consistence of each aspect of involvement. Two items that loaded in more than one factor were excluded from the final involvement profile because of low reliability. Then, a new factor analysis was performed and individual scores were extracted and recorded. Next, hierarchical clustering using Wilk’s lambda agglomeration method was used to cluster the participants according to individual scores extracted from the factor analysis.

Finally, differences between involvement profiles relating to attitude, behavior, beliefs, preferences, extrinsic and intrinsic quality cues, and the sensory perception of light lamb meat were assessed through a one-way analysis of variance with the cluster as the fixed effect.

## 4. Results

### 4.1. Involvement in Light Lamb Meat

The mean values for each item of the involvement scale are shown in Table 1. Because 4 is the neutral point of the scale, in general terms, consumers agree with the items of “hedonic value”, “product importance,” and “risk importance”. However, consumers showed slight disagreement with the items of “symbolic value” and “risk probability”.

The factor analysis summarized the 15 items into 4 factors with eigenvalues higher than 1, explaining 63.1% of the total variability (Table 1). The first factor included the 6 items of two involvement aspects, “product importance,” and “hedonic value”, with a Cronbach’s alpha of 0.80. Therefore, these items were merged into a new aspect of involvement named “pleasure value”. The second most important factor included the aspect of “risk probability” (α = 0.76), and the third factor corresponded to the aspect of “symbolic value” (α = 0.67). The items in “risk importance” had low reliability, and two of the three items were removed from the final involvement profile because they loaded on different factors.

### 4.2. Involvement-based Segmentation of Consumers

**Figure 2:**
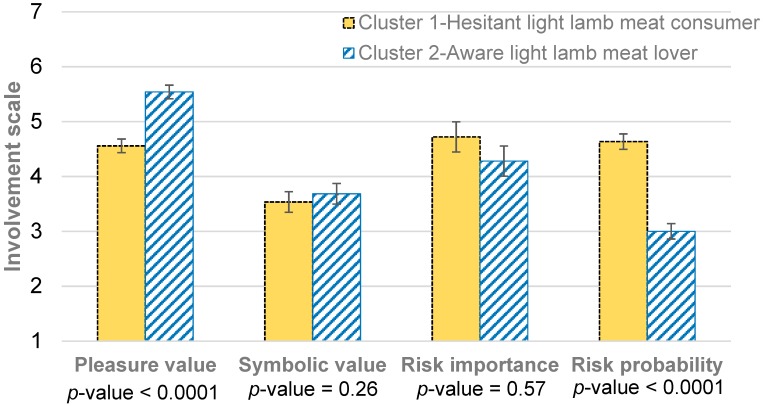
Involvement-Based Segmentation of Consumers: Cluster 1—hesitant light lamb meat consumer (43%) and Cluster 2—aware light lamb meat lover (57%). Each item was scored on a seven-point Likert scale, ranging from 1 = “totally disagree” to 7 = “fully agree”.

The cluster analysis classified light lamb meat consumers into homogenous groups based on their profiles of involvement in light lamb meat. Two clusters optimized the intracluster and intercluster variance. The scores of the aspects of involvement for both clusters are shown in Figure 2. Differences between clusters were significant for “pleasure value” and “risk probability” (*p*-value < 0.001), while there were no differences for “symbolic value” and “risk importance” (*p*-value > 0.05). The first cluster had a lower “pleasure value” (4.56) and a higher “risk probability” than the other cluster (5.54 and 3.00, respectively). Both clusters scored 3 to 5 points for the “symbolic value” and the “risk importance”; these results verified H_1_, so it is confirmed that in Aragón, consumers have a high involvement in light lamb meat.

Accordingly, the first cluster grouped consumers who enjoyed eating light lamb meat, were conscious of their self-image, and perceived the consequences of a poor choice. Another distinctive characteristic of these consumers is that they perceived the probability of making an incorrect choice as high. Therefore, this cluster grouping of 43% of the consumers can be called “hesitant light lamb meat consumers”. The second cluster grouped consumers who truly loved eating lamb meat were also conscious of their self-image and perceived the consequences of a poor choice. These consumers were quite sure that they do not make incorrect choices. Although both involvement-based profiles showed high involvement in light lamb meat, we can conclude that the “aware light lamb meat lovers” had higher involvement than the “hesitant light lamb meat consumers”. Therefore, this cluster grouping of 57% of the consumers can be called “aware light lamb meat lovers”. These results confirm the second hypothesis (H_2_), which refers to consumers being grouped according to their involvement in light lamb meat.

There was no difference in gender between the clusters (χ^2^ = 0.24; *p*-value = 0.6), and the percentage of women in the general sample was 54%. The average age was 48 (S.D. = 12.9), with no differences between the clusters (χ^2^ = 38.9; *p*-value = 0.6). In addition, there were no differences between clusters regarding the representation of families with children (χ^2^ = 0.007; *p*-value = 0.9), living environment (χ^2^ = 3.1; *p*-value = 0.2), level of studies (χ^2^ = 2.1; *p*-value = 0.4), and the frequency of light lamb meat consumption (χ^2^ = 5.3; *p*-value = 0.150). Of the consumers, 36% reported eating light lamb meat less than 1 day per week, 60% ate light lamb meat between 1 to 3 days per week, and the remaining 4% ate light lamb meat more than 3 days per week.

### 4.3. Consumer Attitude and Concerns

Consumers were asked about several concerns related to health and meat consumption. The results of the analysis of variance are shown on Table 2. The Cronbach’s alpha of the items was 0.399, demonstrating that there was no single construct. Both clusters were similarly concerned about the use of antibiotics and hormones, as well as fat, cholesterol, and colorectal cancer (*p*-value > 0.05). Both clusters showed a slight preference for healthy rather than tasty food, but the scores were different (*p*-value = 0.022). Therefore, the “hesitant light lamb meat consumers” agreed slightly more with the proposed preference for tasty over healthy food (3.86) than did the “aware light lamb meat lovers” (3.09). Consequently, these results did not support H_3_ (Highly involved consumers have fewer concerns related to health than less involved consumers).

### 4.4. Consumer Behavior

The involvement-based profiles showed differences in behavioral outcomes, such as modifications of past, present, and future consumption. “Aware light lamb meat lovers” declared that they did not decrease their consumption from the past (*p*-value = 0.042), are increasing their current consumption (*p*-value < 0.001), and that they will not decrease their consumption in the near future (*p*-value = 0.028), compared with the “hesitant light lamb meat consumers”. Hence, the results clearly confirmed H_4.1_ because highly involved consumers do not modify their past or future frequency of consumption, while less involved consumers do.

In general, consumers preferred to purchase light lamb meat sliced at the time of purchase over meat that is packaged in trays or quarters. However, “aware light lamb meat lovers” purchased more quarters (*p*-value = 0.036) and sliced meat (*p*-value = 0.001) than “hesitant light lamb meat consumers”. Both groups made choices regarding the purchase of meat based on price without differences between them (*p*-value = 0.692). Therefore, the hypothesis H_4.2_ (less involved consumers prefer the convenience format of light lamb meat) is partially rejected because of the similar preference for trays.

### 4.5. Consumer Beliefs

Independent of cluster (*p*-value > 0.2), consumers highly disagreed with the belief that light lamb meat is for children and women and dark-colored light lamb meat is better than pale-colored light lamb meat. However, both clusters highly agreed with the belief that light lamb meat is healthy.

“Aware light lamb meat lovers” agreed more with the beliefs that pale meat comes from young lambs (*p*-value = 0.020) and that light lamb meat with yellow fat is worse than meat with white fat (*p*-value = 0.022) compared to “hesitant light lamb meat consumers”. The latter tended to think that light lamb meat has a strong taste/smell, although the difference was not statistically significant (*p*-value = 0.067).

Therefore, hypothesis H_5.1_ (consumers agreed with the proposed beliefs) was almost completely rejected, while hypothesis H_5.2_ (highly involved consumers show a higher degree of agreement with the proposed beliefs than less involved consumers) was partially confirmed.

### 4.6. Consumer Preferences

There were no differences between clusters regarding the questions about preferences (*p*-value > 0.05). In general, consumers did not like fatty light lamb meat. In addition, consumers reported neutral scores regarding liking pale light lamb, white fat, and a preference for red, rather than pink, light lamb meat.

### 4.7. Extrinsic and Intrinsic Quality Cues

**Figure 3:**
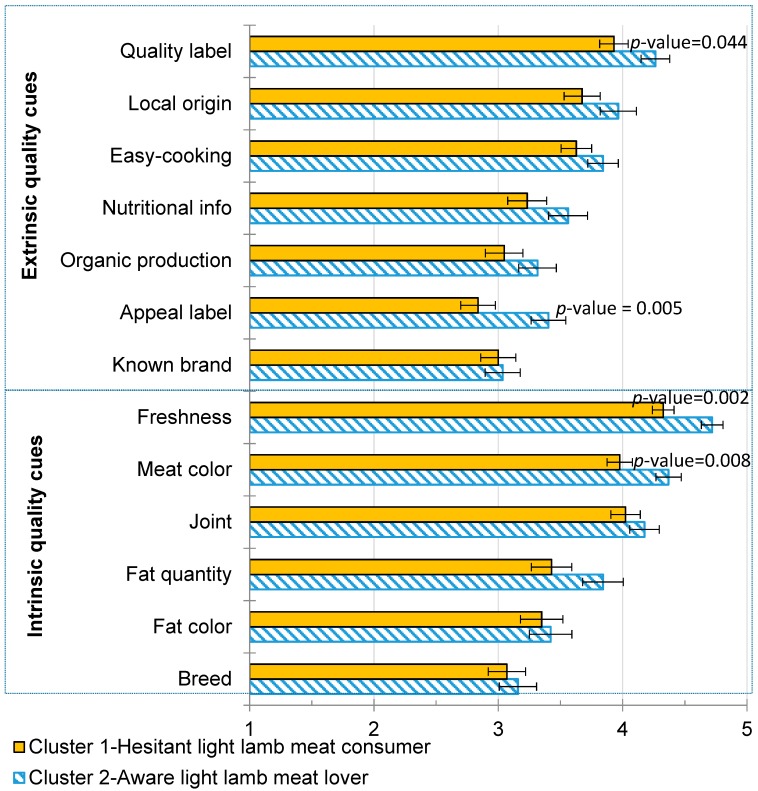
Effect of involvement on the importance of extrinsic and intrinsic quality cues. Quality cues were scored on a five-point scale, in which 1 = “none or very little importance”, 2 = “little importance”, 3 = “average importance”, 4 = “quite a lot of importance,” and 5 = “great importance”.

The results of the analysis of variance of extrinsic and intrinsic quality cues are shown in Figure 3. The most-valued quality cues (approximately 4, “quite a lot of importance”) were “freshness”, “meat color,” and “joint” for the intrinsic cues, and “quality label” for the extrinsic cues (Figure 3). Both involvement-based profiles scored similarly regarding the average importance of extrinsic and intrinsic quality cues (*p*-value = 0.63). Therefore, the averaged means of the extrinsic and intrinsic quality cues were 3.5 and 3.9, respectively (*p*-value < 0.001). Consumers placed more importance on intrinsic than on extrinsic cues; for that reason, hypothesis H_6.1_ (highly involved consumers trust extrinsic cues more than intrinsic cues) was rejected.

The “aware light lamb meat lovers” assigned more importance to the “quality label” such as those including PGI and PDO (*p*-value = 0.044), “appeal of the label” (*p*-value = 0.005), “freshness” (*p*-value = 0.002), and “meat color” (*p*-value = 0.008) compared to the “hesitant light lamb meat consumers”. Other quality cues were scored similarly by both involvement profiles (*p*-value > 0.05). Therefore, the averaged importance of the quality cues for the “hesitant light lamb meat consumers” was lower (3.6) than that for the “aware light lamb meat lovers” (3.8). For this reason, H_6.2_ (highly involved consumers give more importance to quality cues than less involved consumers) was partially accepted because, while some cues were more important for the “aware light lamb meat lovers,” most were found to have no difference between the profiles.

### 4.8. Sensory Analysis of Light Lamb Meat

Since consumers scored similarly for both presented samples (*p*-value > 0.05), the mean of both samples was used to compare the perception of sensory characteristics between the two involvement-based profiles (Table 3). There were no differences between the profiles regarding their preference for juiciness (*p*-value = 0.46), flavor (*p*-value = 0.39), and tenderness (*p*-value = 0.17). Therefore, the seventh hypothesis (H_7_), which hypothesized that sensory perception of light lamb meat is modified by involvement, was rejected.

## 5. Discussion

Involvement is a multidimensional construct. In the study by Laurent and Kapferer [11], the methodology is based on the five aspects of consumer involvement in a certain food product. However, this number is frequently reduced; in the same paper [11], which examines involvement in several food and nonfood products, the “importance of product” and “risk importance” were merged into a new and reliable aspect. In the present study, “product importance” and “hedonic value” could be merged into a new aspect, which aligns with the studies of Verbeke and Vackier [12] and Borgogno, Favotto, Corazzin, Cardello, and Piasentier [14], which both researched involvement in fresh meat. The factor called “pleasure value” explained most of the variability in the previously mentioned studies. The importance of light lamb meat and fresh meat, as perceived by the consumer, is strongly related to consumers’ enjoyment when meat products are consumed. Despite the differences between the involvement-based profiles, the most important aspect of meat is the “pleasure value” and the “risk importance,” while the “symbolic value” and the “risk probability” of fresh meat and light lamb meat are low (scores < 4) [12,14]. Schulz and Hamm [13] did not report the values of the involvement aspects in beef, but they found significant differences between three profiles of involvement (low, medium, high). These authors posited the importance of the “hedonic value,” “importance of product,” and “risk importance” aspects and showed that “symbolic value” and “risk probability” cannot be used to explain the differences between involvement-based profiles. The consumers from Aragón showed higher scores for “symbolic value” than those reported by Verbeke and Vackier [12], but the scores were similar to those reported Borgogno, Favotto, Corazzin, Cardello, and Piasentier [14]. In addition, the “risk importance” was high, demonstrating that light lamb meat is perceived by consumers as a high involvement product because they perceive the consequences of making an incorrect choice as very undesirable [12]. Beharrell and Denison [18] compared the purchasing of fresh meat with other food products such as soup, cereals and bakery items and found that Spanish consumers were highly involved in meat. The importance given to the correct choice at the time of purchase was important and comparable to buying car insurance or restaurant visits [65]. Therefore, it has been demonstrated that light lamb meat is not a low involvement food product in the region of study. However, as shown by Verbeke and Vackier [12], fresh meat in general, as well as light lamb meat, cannot be dually classified as a low or high-involvement product; it is the consumer who is either more or less involved in light lamb meat.

Verbeke and Vackier [12] found four fresh-meat market segments with sociodemographic differences. However, the profiled consumers in our study were only different regarding their involvement, and the sociodemographic variables were unimportant. These results support previous research findings, showing that when consumers are grouped by homogeneous types according to their food-related lifestyles, perceptions or other psychographic variables, the sociodemographic variables are often less important [10,66,67,68]. The lack of difference in sociodemographic variables may be the consequence of the generalized high consumption of light lamb meat in the studied region. Therefore, the classic association between women and low consumption of red meat [69,70,71] is not evident for light lamb meat in our study. These results also were reported previously by Escriba-Perez, Baviera-Puig, Buitrago-Vera, and Montero-Vicente [68].

Health concerns are an important factor in the choice of food and an important determinant of attitude [72]. Studies have reported that an important percentage of the population (28%) was considering a reduction in meat consumption [73]. In this study, the most important attitude driving the decrease in meat consumption was health. However, the importance given to health concerns depends on the consumer [74]. Therefore, the consumers highly involved in fresh meat, “straightforward meat lovers,” in the study by Verbeke and Vackier [12], and “aware light lamb meat lovers” in our study, did not reduce their consumption; in fact, these groups were observed to increase their consumption. Conversely, less involved consumers are also prone to modifying their meat consumption due to meat-related security crises (BSE, dioxins, and colorectal cancer). In contrast, Schulz and Hamm [13] reported that consumers with a high involvement in beef decreased their beef consumption during the BSE crisis. This observation could be explained in two ways. First, the temporal distance between the beef crises, as indicated by Schulz and Hamm, and the other reason could be that even though the IARC [38] related colorectal colon to red meat, consumers did not think that light lamb meat is a type of red meat. However, inconsistences between the attitude and behavior of consumers have been previously detected by social psychological research [75]. These inconsistences could explain the discrepancy between beliefs (“light lamb meat is healthy”), attitudes (“healthy eating is more important than enjoyment”), and behavior (e.g., “decreasing consumption of meat”).

The purchasing choices of highly involved consumers are scarcely driven by the price of meat, as they reported a low response to bargains [12,13]. However, both involvement profiles in our study showed that price has an important influence on their choices. In agreement with this finding, Ripoll et al. [76] reported that most of the consumers grouped according to their food-related lifestyles thought that the price of light lamb is very important. Price is an important factor when imported lamb, having a higher slaughter weight and different intrinsic qualities, appears on the market. Highly involved consumers would be less prone to confuse the meat because they base their product evaluations on intrinsic cues. However, less-involved and uninformed consumers could purchase such imported meat on the basis of its low price, because they are less motivated to make an effort in the decision-making process [22,29]. This phenomenon could be taken into account when driving initiatives to increase consumption, because less involved consumers pay less attention to media and information services. In terms of food-related lifestyles, Witzling and Shaw [77] found that food-related messages are unlikely to reach uninvolved consumers.

Regarding the form of presentation, consumers highly involved in beef more commonly purchase meat from a butcher [13]. It seems that consumers do not like excessive handling of meat, showing a preference for fresh-cut meat at the time of purchase [48]. However, there are consumers that prefer light lamb meat packed in trays because they look for convenient packaging and a form that is easily cooked [10]. These “uninvolved” and “careless” consumers differ from other consumers because they are more receptive to convenience products and place less importance on intrinsic quality cues in general [10], in agreement with the findings of our study. The results of these authors also agree with our results regarding the influence that quality cues have on consumers’ perceived risk, which differs between more and less involved consumers [28]. Therefore, the less involved consumers reported that quality cues were less important and more often perceived a high-risk probability than the more involved consumers. When the importance of cues is not influenced by environment, it has been demonstrated that the intrinsic quality cues of light lamb are more valuable than extrinsic cues. However, some authors report that in retail environments, consumers use extrinsic more than intrinsic quality cues [78], because the latter cannot be assessed when buying. Therefore, the consumer importance toward risk increases [79]. It has been shown that the presence of a quality label (PGI, PDO) was more important for “aware light lamb meat lovers” than for “hesitant light lamb meat consumers”. Espejel, Fandos, and Flavián [28] argue that consumers’ involvement level with food products that have a quality label may also introduce remarkable changes in consumer behavior because the quality label increases the product’s symbolism. In fact, 78% of consumers reported that light lamb meat labeled with a mark of quality is better than other light lamb meat [62]. In addition, the purchase of food products with a quality label reduces the perceived risk by consumers of making a mistake that might have health implications [28]. In addition, when consumers perceive high risks, they tend to rely heavily on only a few cues, especially the brand [41]. As a result, both our groups of consumers gave low importance to brand because both had a relatively high involvement. Relating the preference of “aware light lamb meat lovers” for unpacked meat to the credence quality attributes or beliefs and the high use of intrinsic cues, Becker [42] reported that the consumer can screen quality by a visual inspection of the unpacked food product and additional information that is supplied by the sales staff. Therefore, these “aware light lamb meat lovers” are prone to agree with some beliefs and use intrinsic cues extensively. As reported in our study, the most important intrinsic quality cues were color and freshness when assessing expected quality [48]. Bernués, Ripoll, and Panea [10] reported that freshness was also the most important intrinsic cue for every group of consumers but that color was a less important cue for “uninvolved” consumers.

The consumer sensory acceptance of certain meats and cheeses is driven by familiarity and involvement, even in blinded conditions. In general, high familiarity and involvement leads to high acceptance and preference scores [14,56,62]. However, both groups of consumers similarly scored the meat in blinded conditions without interference from the environment. In general, highly involved consumers were highly familiar with the product and had a higher consumption than the less involved consumers. Frequency of consumption and repeated exposure can influence the sensory perception [80,81]; however, both groups in our study generally had an important involvement and similar consumption of light lamb meat, which could explain the lack of difference.

## 6. Conclusions

This study focuses on the enduring involvement in a food product by consumers in a high-consumption region. The results suggest that consumers in the studied region were highly involved in light lamb meat; however, differences are due to the perceived importance of the product together with the hedonic value and the risk probability of making a mistake. Therefore, the two different groups of consumers had different behaviors and some different beliefs. In addition, the levels of involvement did not influence most of the health-related attitudes, preferences, or sensory perceptions related to light lamb meat. To validate our results, it would be useful to replicate the study in another Spanish region with high or low levels of consumption. It would also be useful to study the situational involvement or consumer involvement in a purchasing environment. Although the light lamb consumption in Aragón is decreasing, the consumption still remains high. Future studies may extend the analysis to territories with intense decrease in lamb meat consumption to better understand the consumers’ involvement in that context.

Regarding sensory analysis, the sample size might not have been large enough to ensure that there are no other differences between the groups of consumers. However, according to Hough et al. [82], the number of consumers was sufficient to perform an acceptability test with a 9-point scale. As mentioned, the involvement of consumers had a great influence on their reaction to food-related information. Therefore, it would be interesting to understand the effects of mass media and advertisements on these consumers by adding the factors of trust and extensiveness toward the decision-making dimensions of the methodology developed in Laurent and Kapferer [11]. This knowledge could be a useful tool for directing the efforts of the meat market in preventing the continuing decrease of light lamb consumption in Spain. Because highly involved consumers are less susceptible to suggestion by advertising campaigns, any action must be oriented to take advantage of the particularities of each group of consumers. Therefore, the hesitant light lamb meat consumers, who are afraid of the probability of making an incorrect choice, can be influenced by advertisements on packaging that reinforce the idea that buying the meat is a guarantee of success. A quality label indicating the origin of the meat could be used, but the use of the particular breed is not interesting. Aware light lamb meat lovers are clearly interested in a product with attractive packaging and quality labels, together with freshness and meat color. Hence, the packaging must be transparent and retain freshness throughout the shelf life of the meat.

## Figures and Tables

**Table 1:** Factor analysis of the 15-item involvement scale (factor loadings from principal component analysis).

Items of Involvement Scale	Mean c	Factor 1	Factor 2	Factor 3	Factor 4
*Product importance*					
I do not care at all about light lamb meat ^a^	5.2	**0.62**			
Light lamb meat is very important to me	4.1	**0.80**			
For me, light lamb meat is absolutely necessary	3.8	**0.68**			
*Hedonic value*					
I can say that I actually do not like to eat light lamb meat ^a^	5.9	**0.65**			
I enjoy a meal with light lamb meat more than a meal without it	4.5	**0.62**			
I appreciate light lamb meat very much	5.2	**0.78**			
*Symbolic value*					
You can tell a lot about a person based on his/her choice of light lamb meat	3.5			**−0.73**	
My choice of light lamb meat gives other people an image of me	3.0			**−0.75**	
My choice of light lamb meat conveys nothing about me to other people ^a^	3.3			**−0.67**	
*Risk importance*					
I do not have a lot to lose when I make a bad choice about light lamb meat ^a,b^	4.6				0.44
I would find a bad choice about light lamb meat terrible	4.5				**0.79**
I find it very annoying to make a wrong choice about light lamb meat ^b^	4.8				0.43
*Risk probability*					
I never know if I make the right choice about light lamb meat	3.5		**0.83**		
When I buy light lamb meat, I know that I make the right choice ^a^	3.6		**0.75**		
I feel lost when having to choose light lamb meat	3.3		**0.81**		
Variability (%)		25.1	16.2	13.3	8.4
% accumulated variability		25.1	41.3	54.7	63.1

^a^ Item reversely scaled. ^b^ These items were not included to extract the individual scores and not used to define the involvement profile and the involvement-based segmentation of consumers (Figure 2). ^c^ Seven-point Likert scale, ranging from 1 = “totally disagree” to 7 = “fully agree”. Factor loadings in bold denote variables included in the final involvement profile

**Table 2:** Effects of involvement on attitude, behavior, beliefs, and preferences.

Involvement-Related Items	Aware Light Lamb Meat Lover	Hesitant Light Lamb Meat Consumer	s.e.	*p*-Value
*Attitude*				
Concern about antibiotics/hormones	5.95	5.53	0.20	0.16
Concern about fat/cholesterol	5.37	5.44	0.23	0.82
Concern about cancer of colon	4.21	4.37	0.26	0.67
What I like to eat is more important than healthy eating	3.09	3.86	0.24	0.02
*Behavior*				
Decreased consumption from the past	3.37	4.02	0.22	0.04
Increasing consumption	4.14	3.26	0.17	0.001
Intention to decrease in near future	2.39	3.02	0.20	0.03
Format of purchase: quarters	4.23	3.53	0.23	0.04
Format of purchase: packaged in trays	3.47	3.72	0.26	0.5
Format of purchase: sliced at time of purchase	5.54	4.58	0.20	0.001
It is mainly the price that determines my choice of meat	4.09	3.98	0.20	0.69
The geographical origin of light lamb is important	5.89	5.37	0.18	0.04
Only eat light lamb meat at celebrations	2.79	3.14	0.22	0.26
*Beliefs*				
Light lamb meat is food for children and women	2.32	2.05	0.22	0.40
Dark colored lamb meat is better than pale	3.21	3.35	0.17	0.57
Light lamb meat is healthy	5.46	5.12	0.19	0.22
Pale meat comes from young lambs	4.51	3.91	0.18	0.02
Light lamb meat with yellow fat is bad quality	4.46	3.88	0.17	0.02
Light lamb meat has strong taste/smell	3.54	4.14	0.23	0.07
*Preferences*				
I like fatty light lamb meat	2.72	2.79	0.20	0.80
I like pale light lamb meat	4.53	4.21	0.18	0.22
I like light lamb meat with white fat	4.28	3.81	0.19	0.09
I like red light lamb meat rather than pink	4.07	4.19	0.20	0.68

Each item was scored on a seven-point Likert scale, ranging from 1 = “totally disagree” to 7 = “fully agree”.

**Table 3:** Effect of involvement on acceptability of sensory parameters.

Sensory Parameters	Aware Light Lamb Meat Lovers	Hesitant Light Lamb Meat Consumers	s.e.	*p*-Value
Juiciness ^↑^	6.2	6.0	0.11	0.46
Flavor ^↑^	6.4	6.1	0.15	0.39
Tenderness ^↑^	6.6	6.2	0.19	0.17

^↑^ 9-point hedonic scale ranging from 1 (dislike extremely) to 9 (like extremely).
